# Optimization of ultrasound-assisted extraction of flavonoid compounds and their pharmaceutical activity from curry leaf (*Murraya koenigii* L.) using response surface methodology

**DOI:** 10.1186/1472-6882-14-318

**Published:** 2014-08-28

**Authors:** Ali Ghasemzadeh, Hawa ZE Jaafar, Ehsan Karimi, Asmah Rahmat

**Affiliations:** Department of Crop Science, Faculty of Agriculture, University Putra Malaysia, 43400 Serdang, Selangor Malaysia; Department of Nutrition & Dietetics, Faculty of Medicine & Health Sciences, University Putra Malaysia, 43400 UPM Serdang, Selangor Malaysia

**Keywords:** Response surface methodology, Ultra high performance liquid chromatography, Ultrasound-assisted, Curry leaf, HeLa cancer, Flavonoids

## Abstract

**Background:**

Extraction prior to component analysis is the primary step in the recovery and isolation of bioactive phytochemicals from plant materials.

**Methods:**

Response surface methodology was applied to optimize ultrasound-assisted extraction conditions followed by ultra high performance liquid chromatography (UHPLC) to achieve high catechin, myricetin, and quercetin contents, and high antioxidant and anticancer activities in the curry leaf extracts. The antioxidant and anticancer activities of the leaf extracts were determined by the 1,1-diphenyl-2-picryl-hydrazyl (DPPH) and 3-(4,5-dimethylthiazol-2-yl)-2,5-diphenyltetrazolium bromide (MTT) assays, respectively. The central composite experimental design (3-level, 3-factorial) was employed to consider the effects of ultrasonic power (80–150 W), temperature (40–80°C), and methanol dilution (40–80%) on the properties of the curry leaf extracts.

**Results:**

It was found that ultrasonic power of 145.49 W at 55.9°C with 80% methanol was the most appropriate set of conditions for the extraction of catechin, myricetin, and quercetin from curry leaves with the consequent high antioxidant activity. Using the optimum extraction conditions, the extraction yields of catechin, myricetin, and quercetin were 0.482, 0.517, and 0.394 mg/g DW, respectively, and the antioxidant activity was enhanced to 83%. The optimized extract showed more distinct anticancer activity against HeLa cancer cells in a concentration of 67.2 μg/mL (*P* < 0.01) without toxicity to normal cells.

**Conclusions:**

The results indicated that the pharmaceutical quality of curry leaves could be improved significantly by optimizing the extraction process using response surface methodology.

## Background

Medicinal plants are the richest source of bioactive compounds used in traditional and modern medicine [1]. Flavonoids and phenolics are essential groups of plant phytochemicals with superoxide radical scavenging activity, thereby providing anticancer activity [2, 3]. In the herbal medicine industry, the extraction process is the important step for the isolation of phytochemicals from herbs and spices [4]. Extraction of herbs using an ultrasound-assisted process was recommended previously as a one of the most inexpensive and simplest existing extraction systems, and could be suitably operated rapidly for large-scale preparations [5]. The application of ultrasound helps develop interesting and novel methodologies in food processing; these methodologies are often complementary to classical methods [6]. Ultrasound-assisted extraction can accelerate heat and mass transfer, has been successively used in the extraction field, and is well known to have a significant effect on the rate of various processes in the food industry [7]. Ultrasound waves interact with the plant material and alter its physical and chemical properties; furthermore, their cavitational effect facilitates the release of extractable compounds and enhances the mass transport by disrupting the plant cell walls [7]. Previous studies have demonstrated that the extraction yield of flavonoid compounds depends strongly on the extraction technique, solvent polarity, and temperature [8, 9]. A developed model is required for optimizing the independent variables in order to get superior extraction yields from herbs. Response surface methodology (RSM) is a collection of statistical and mathematical techniques that are used to optimize the range of variables in various experimental processes to reduce the number of experimental runs, cost, and time, compared to other methods [10, 11]. *Murraya koenigii* (L.), generally known as the curry leaf, or Pokok kari (Daun kari) in Malaysia, is one of the traditional folk remedies that contains several interesting bioactive compounds [12] with anti-tumor [13], antioxidant [14, 15], anti-inflammatory [16], anti-hyperglycemic [17], and hypoglycemic effects [18]. Due to the high beneficial value of this crop, research is required to optimize the extraction process to ensure high nutritional and pharmaceutical quality. However, far too little attention has been paid to the optimization of curry leaf extraction in folk medicine. To the best of our knowledge, there have been no studies to optimize the flavonoid extraction from the curry leaf and following that, improvement of the anticancer and antioxidant activities. The current study is designed in order to optimize the ultrasound-assisted extraction conditions of the Malaysian curry leaf (*M. koenigii*) to achieve high flavonoid contents and high antioxidant and anticancer activity by using response surface methodology with a central composite design.

## Methods

### Plant material

Fresh curry leaf samples were obtained from Bachok, Kelantan province, Malaysia. The Malaysian Agriculture Research and Development Institute (MARDI) identified the samples with voucher specimens of MTM0018/1. The leaves were shade-dried (moisture content: 6.2%), powdered (80 mesh), and kept at -20°C.

### Ultrasound-assisted extraction

The curry leaves (1 g) were mixed with methanol (20 mL) with different concentrations from 40% to 80%. The solutions were transferred to an ultrasonic bath. The temperature and ultrasonic power was adjusted from 40 to 80°C and 80 to 150 W, respectively. Extraction was conducted for 20 min under these various conditions. In total, 20 experiments with different variables for extraction were completed (Table 1).

**Table 1:** **Experimental design and observed experimental data**

Treatment	Temperature °C	Methanol concentration %	Ultrasunic power (W)	Catechin	Predicted	Myricetin	Predicted	Quercetin	Predicted	Antioxidant activity	Predicted
1	40	40	80	0.265 ± 0.011	0.267	0.309 ± 0.025	0.311	0.177 ± 0.010	0.179	67.0 ± 2.20	64.6
2(C)	60	60	115	0.426 ± 0.035	0.453	0.470 ± 0.018	0.497	0.338 ± 0.018	0.365	80.0 ± 7.05	79.5
3	40	40	150	0.274 ± 0.014	0.286	0.318 ± 0.021	0.330	0.186 ± 0.008	0.198	66.2 ± 4.72	65.4
4	80	80	80	0.191 ± 0.002	0.233	0.235 ± 0.017	0.277	0.103 ± 0.011	0.145	52.0 ± 7.76	55.2
5 (C)	60	60	115	0.396 ± 0.005	0.453	0.440 ± 0.016	0.497	0.308 ± 0.017	0.365	79.5 ± 2.30	79.5
6 (C)	60	60	115	0.436 ± 0.017	0.453	0.480 ± 0.007	0.497	0.348 ± 0.019	0.365	77.0 ± 3.82	79.5
7	80	40	150	0.234 ± 0.008	0.247	0.278 ± 0.014	0.291	0.146 ± 0.009	0.159	55.4 ± 1.81	57.19
8	80	40	80	0.222 ± 0.012	0.247	0.266 ± 0.011	0.291	0.134 ± 0.014	0.159	55.0 ± 2.30	55.4
9	40	80	150	0.347 ± 0.011	0.376	0.391 ± 0.014	0.420	0.259 ± 0.012	0.288	71.0 ± 3.01	73.14
10	40	80	80	0.295 ± 0.007	0.335	0.339 ± 0.017	0.380	0.207 ± 0.011	0.247	69.0 ± 5.56	69.75
11	80	80	150	0.204 ± 0.019	0.255	0.244 ± 0.006	0.296	0.116 ± 0.007	0.167	54.7 ± 4.12	59.59
12(C)	60	60	115	0.474 ± 0.010	0.453	0.518 ± 0.026	0.497	0.386 ± 0.016	0.365	78.0 ± 2.69	79.5
13	86.32	60	115	0.188 ± 0.015	0.213	0.232 ± 0.019	0.247	0.100 ± 0.011	0.105	51.0 ± 1.88	53.5
14(C)	60	60	115	0.466 ± 0.008	0.453	0.510 ± 0.013	0.497	0.378 ± 0.017	0.365	80.4 ± 3.27	79.5
15	60	60	161.06	0.480 ± 0.017	0.454	0.524 ± 0.019	0.498	0.392 ± 0.020	0.366	83.0 ± 4.61	79.7
16	60	86.32	115	0.536 ± 0.014	0.464	0.580 ± 0.018	0.507	0.448 ± 0.015	0.376	87.0 ± 3.55	84.8
17	60	60	68.93	0.456 ± 0.018	0.434	0.500 ± 0.015	0.478	0.368 ± 0.013	0.346	75.0 ± 2.70	77.1
18	60	33.67	115	0.403 ± 0.018	0.426	0.447 ± 0.008	0.470	0.315 ± 0.010	0.338	73.0 ± 3.29	75.2
19	33.67	60	115	0.244 ± 0.014	0.285	0.288 ± 0.017	0.317	0.156 ± 0.016	0.188	58.0 ± 1.77	60.9
20(C)	60	60	115	0.460 ± 0.010	0.453	0.504 ± 0.017	0.497	0.372 ± 0.019	0.365	79.5 ± 2.11	79.5

Catechin, myricetin and quercetin identified by UHPLC and antioxidant activity was measured by DPPH assay.

(C): central points; Catechin, Myricetin and Quercetin content are in mg g^-1^ DW. DPPH activity is in %.

### Identification of flavonoids by Ultra High Performance Liquid Chromatography (UHPLC)

The UHPLC system (Agilent, Model 1200) with a C18 (4.6 × 250 mm, 5 μm) column was used for flavonoid separation and identification. In this system, two mobile phases, 0.03 M orthophosphoric acid (A) and HPLC-grade methanol (B), were used. The column temperature, flow rate, and injection volume were adjusted at 35°C, 20 μL, and 1 mL/min, respectively. The range of the detecting wavelength was between 260 and 360 nm. Gradient elution was performed as follows: 0–10 min for 40–100% B; 10–15 min for 100% B; 15–20 min for 100–40% B, and finally, washing of the column. To prepare the standard solutions, catechin (0.0625, 0.125, 0.250, 0.500, and 1 mg/mL), myricetin (0.031, 0.062, 0.124, 0.248, and 0.496 mg/mL), and quercetin (0.09, 0.18, 0.36, 0.72 and 1.44 mg/mL) were dissolved in the HPLC-grade methanol. The linear regression equation was calculated with *Y* = *aX* ± *b*, where X is the concentration of flavonoid and Y is the peak area of flavonoids obtained from UHPLC.

### 1,1-diphenyl-2-picrylhydrazyl (DPPH) assay

The free radical scavenging activity of the curry leaf extracts was determined according to the method reported by Mensor *et al.* [19] with some modification. DPPH was dissolved in methanol to obtain a final concentration of 2 mM. Then, the DPPH solution (1 mL) was added to different concentrations of curry leaf extracts (20, 40, 60 80, and 100 mg/mL). The mixture was shaken gently and incubated at 28°C in a dark room for 40 min. For the control, methanol was used as a blank. The absorbance of the samples was read at 517 nm using a spectrophotometer. BHT (butylhydroxytoluene) and α-tocopherol were used as positive controls. The scavenging activity was calculated using the following formula:


### Determination of anticancer activity

The frozen cells were retrieved from a liquid nitrogen cell storage tank and thawed rapidly in cryovials. The contents of the cryovial were carefully transferred to a centrifuge tube and a prewarmed media (10 mL) was gradually added to the cell suspension. The centrifuge tube was spun down at 1000 rpm for 10 min and the resulting pellet gently resuspended in fresh media (10 mL) in a culture flask. Subsequent incubation was carried out in a 37°C humidified incubator supplemented with 5% CO_2_. After 24 h, the old medium was discarded one day after seeding and 2–3 mL PBS was added to cover the entire surface and discard. Then, 1.5-2 mL trypsinizing solution was added to cover the flask surface, which was then left at room temperature for 3 min until most of the cells detached. Subsequently, 10 mL of the complete medium was added. For the MTT assay, the cell medium (100 μL) containing various concentrations of the extract (20, 40, 60, 80, 100, and 120 μg/mL) was added into all the wells and incubated in a 37°C, 5% CO_2_ incubator for 72 h. A stock solution of MTT in PBS (5 mg/mL) was prepared and the MTT reagent (20 μL) was added to the cell monolayer. DMSO (dimethyl sulfoxide) (100 μL) was added to each well and mixed thoroughly by pipetting 10–20 times to dissolve the blue formazan crystals. The absorbance of samples was read at 570 nm using an ELISA reader [20].

### Experimental design and statistic analysis

RSM software with the central composite experimental design (3-level, 3-factorial) was used to investigate and validate the extraction parameters affecting the extraction yields of catechin (Y_1_), myricetin (Y_2_), quercetin (Y_3_), and antioxidant activity (Y_4_) of curry leaf extracts. In this study, 20 experiments were designed and carried out in duplicate with different ranges of ultrasonic power, methanol concentration, and extraction temperature, which are presented in Table 1. In order to conduct the experimental design and statistical analysis, Design Expert software (version 6.0) was used. A model was applied to predict the response variables as given below:


Where *Y* is the predicted dependent variable; b_0_ is a constant that fixes the response at the central point of the experiment; *b*_*1*_*, b*_*2*_ and *b*_*3*_ are the regression coefficients for the linear effect terms; *b*_*1*_*b*_*2*_*, b*_*1*_*b*_*3*_ and *b*_*2*_*b*_*3*_ are the interaction effect terms and *b*_*1*_^*2*^*, b*_*2*_^*2*^ and *b*_*3*_^*2*^ are the quadratic effect terms; respectively.

Analysis of variance (ANOVA) and response surface analysis were used to determine the statistical significance of the model. The adequacy of the model was predicted through the ANOVA (*P* < 0.05) and regression analysis (R^2^). The relationship between the response and independent variables was demonstrated using a response surface plot.

## Results and discussion

### Statistical significance analysis, response surface and model fitting of catechin extraction

**Figure 1:**
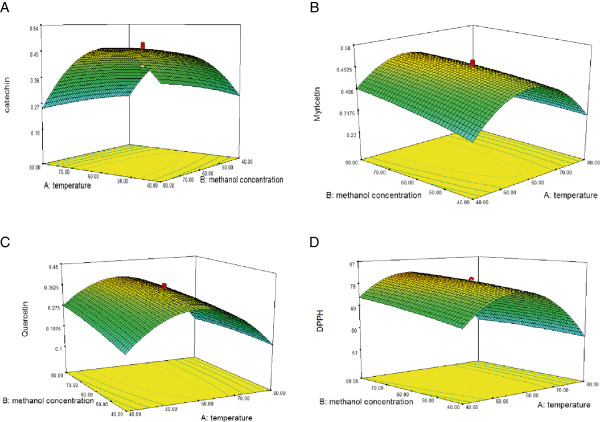
**Effect of methanol concentration (%) and temperature (°C) on the extraction yield of catechin (A), myricetin (B), and quercetin (C), and antioxidant activity (D) at an ultrasonic power of 115 W.**

The results of the response surface methodology demonstrated significant (*P* < 0.05) regression relationships between catechin and independent variables. The highest and lowest contents of catechin in the curry leaf extracts were obtained using treatments 12 and 13, respectively, with the respective values of 0.477 and 0.188 mg/g DW (Table 1). The results indicated significant (*P* < 0.01) quadratic and linear effects of the extraction temperature on catechin content (Table 2). The value of catechin decreased by about 12.8% when the concentration of methanol increased from 40% to 80% (80°C, 150 W) (Figure 1A). The yield of catechin attained the peak value (0.536 mg/g DW) at 86.3% methanol concentration at an extraction temperature of 60°C. Increasing the ultrasonic power from 80 to 150 W increased the content of catechin by about 6% (80°C, 80% methanol). The predicted model developed for catechin (Y_1_) was as follows:

**Table 2:** **ANOVA for response surface models of all independent variables**

Factor	Catechin		Myricetin		Quercetin		DPPH	
	Mean	p-value	Mean	p-value	Mean	p-value	Mean	p-value
	Square	Prob > F	Square	Prob > F	Square	Prob > F	Square	Prob > F
Model	0.0225	< 0.0001**	0.0226	< 0.0001**	0.0225	< 0.0001**	244.3220	0.0001**
Linear term								
X_1_	0.0142	0.0060**	0.0145	0.0061**	0.0142	0.0060**	372.0941	0.0006**
X_2_	0.0041	0.0873	0.0040	0.0954	0.0041	0.0873	40.4155	0.1258
X_3_	0.0012	0.3259	0.0011	0.3466	0.0012	0.3259	19.1805	0.2749
Quadratic								
X_1_^2^	0.1637	< 0.0001**	0.1640	< 0.0001**	0.1637	< 0.0001**	1599.0550	< 0.0001**
X_2_^2^	0.0005	0.5309	0.0005	0.5285	0.0005	0.5309	2.2023	0.7028
X_3_^2^	0.0006	0.4902	0.0006	0.4883	0.0006	0.4902	8.9640	0.4472
Interactions								
X_1_X_2_	0.0034	0.1168	0.0035	0.1126	0.0034	0.1168	13.7813	0.3502
X_1_X_3_	0.0002	0.7122	0.0002	0.6853	0.0002	0.7122	0.4513	0.8624
X_2_X_3_	0.0002	0.6527	0.0002	0.6853	0.0002	0.6527	3.2513	0.6436

Independent variable X_1_: Temperature, X_2_: Mthanol concentration, X_3_: Ultrasonic power; **Significant at *P* < 0.01.

Analysis of variance for predicted models implied that the model was highly significant (F-value 20.13, *P* < 0.0001) with a good coefficient of determination (R^2^ = 0.98). In addition, lack-of-fit (F-value 1.77, *P* > 0.05) was not significant (Table 3). For a good model development by RSM, although the lack-of-fit must not be significant, the model must be significant [21].

**Table 3:** **Statistical parameters calculated after implementation of 2nd full factorial central composite experimental design**

Regression coefficient	R ^2^	R ^2^ (adjusted)	F-value of model	Regression ( *P-*value)	Lack of fit (F-value)	Lack of fit ( *P*-value)
Y_1_	0.98	0.97	20.13	0.0001	1.77	0.299
Y_2_	0.97	0.95	19.77	0.0001	1.85	0.289
Y_3_	0.99	0.99	21.48	0.0001	1.94	0.274
Y_4_	0.99	0.99	17.21	0.0001	1.61	0.261

### Statistical significance analysis, response surface and model fitting of myricetin extraction

The extraction of myricetin was significantly (*P* < 0.01) influenced by the temperature. The highest and lowest contents of myricetin in the curry leaf extracts were obtained using treatments 16 and 13, respectively, with the respective values of 0.58 and 0.233 mg/g DW. On increasing the temperature from 40 to 56°C (80% methanol 150 W), the myricetin content increased by about 24.6%. On further increasing the extraction temperature from 56 to 80°C, similar to the trend observed for catechin, the content of myricetin decreased significantly by about 37.5% (Figure 1B). Increasing the ultrasonic power from 80 to 150 W increased the content of myricetin in the extract by about 3.8% (80°C, 80% methanol), but no significant effect was found. The predicted model obtained for myricetin (Y_2_) was as follows:


Analysis of variance for predicted models implied that the model was highly significant (F-value 19.77, *P* < 0.0001) with a good coefficient of determination (R^2^ = 0.97). In addition, lack-of-fit (F-value 1.85, *P* > 0.05) was not significant.

### Statistical significance analysis, response surface and model fitting of quercetin extraction

**Figure 2:**
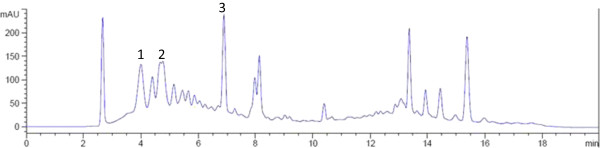
**UHPLC chromatogram of curry leaf extract.** Identified compounds are: catechin (1), myricetin (2) and quercetin (3).

The highest and lowest contents of quercetin in the curry leaf extracts were obtained using treatment 16 and 13, respectively, with the respective values of 0.448 and 0.1 mg/g DW (Table 1). The extraction temperature had significant (*P* < 0.01) quadratic and linear effects on the quercetin content, whereas the effect of methanol concentration and ultrasonic power was not significant. As shown in Figure 1C, the quercetin content increased with increasing methanol concentration. Increasing the temperature from 40 to 58°C enhanced the quercetin content; however, further increasing the temperature to 80°C led to a significant decrease in the quercetin content. The predicted model obtained for the extraction yield of quercetin (Y_3_) was as follows:


Analysis of variance for predicted models implied that the model was highly significant (F-value 21.48, *P* < 0.0001) with a good coefficient of determination (R^2^ = 0.99). In addition, lack-of-fit (F-value 1.94, *P* > 0.05) was not significant (Table 3). The UHPLC chromatogram of the identified flavonoid compounds from the curry leaf extract is shown in Figure 2.

Degradation of the flavonoid and phenolic compounds has been observed with the use of high temperatures [22]. In addition, the Maillard reaction may occur at high temperatures, forming undesired compounds [23, 24]. Decreasing the flavonoid concentration in the extract at high temperatures by ultrasound-assisted extraction could be related to the degradation of these compounds at higher temperatures (>56°C). Pingret *et al.* [25] reported that the formation of degradation products increased owing to the treatment performed with a titanium horn in the ultrasound process. Chemat *et al.* [6] demonstrated that the flavor and composition of some edible oils such as hexanal and limonene are deteriorated by the ultrasound treatment (150 W).

### Statistical significance analysis, response surface and model fitting of antioxidant activity

As shown in Table 2, the DPPH activity of all extracts was more than 51%. The DPPH activity of the extract ranged from 51% to 87% when treatments 13 and 16 were employed, respectively. Independent variables showed significant effect on the antioxidant activity of the extracts with a good regression equation (*P* < 0.05, R^2^ = 0.99) (Table 3). The antioxidant activity decreased in the curry leaf extracts on increasing the temperature from 56 to 80°C, decreasing the methanol concentration from 80% to 40%, and decreasing the ultrasonic power from 150 to 80 W (Figure 1D). The extraction variables showed significant (*P* < 0.01) quadratic and linear effects on the antioxidant activity (Table 2). The predicted model obtained for DPPH (Y_4_) was as follows:


Analysis of variance for predicted models implied that the model was highly significant (F-value 17.21, *P* < 0.0001) with a good coefficient of determination (R^2^ = 0.98). Moreover, lack-of-fit (F-value 1.61, *P* > 0.05) was not significant (Table 3).

### Optimization of responses

**Figure 3:**
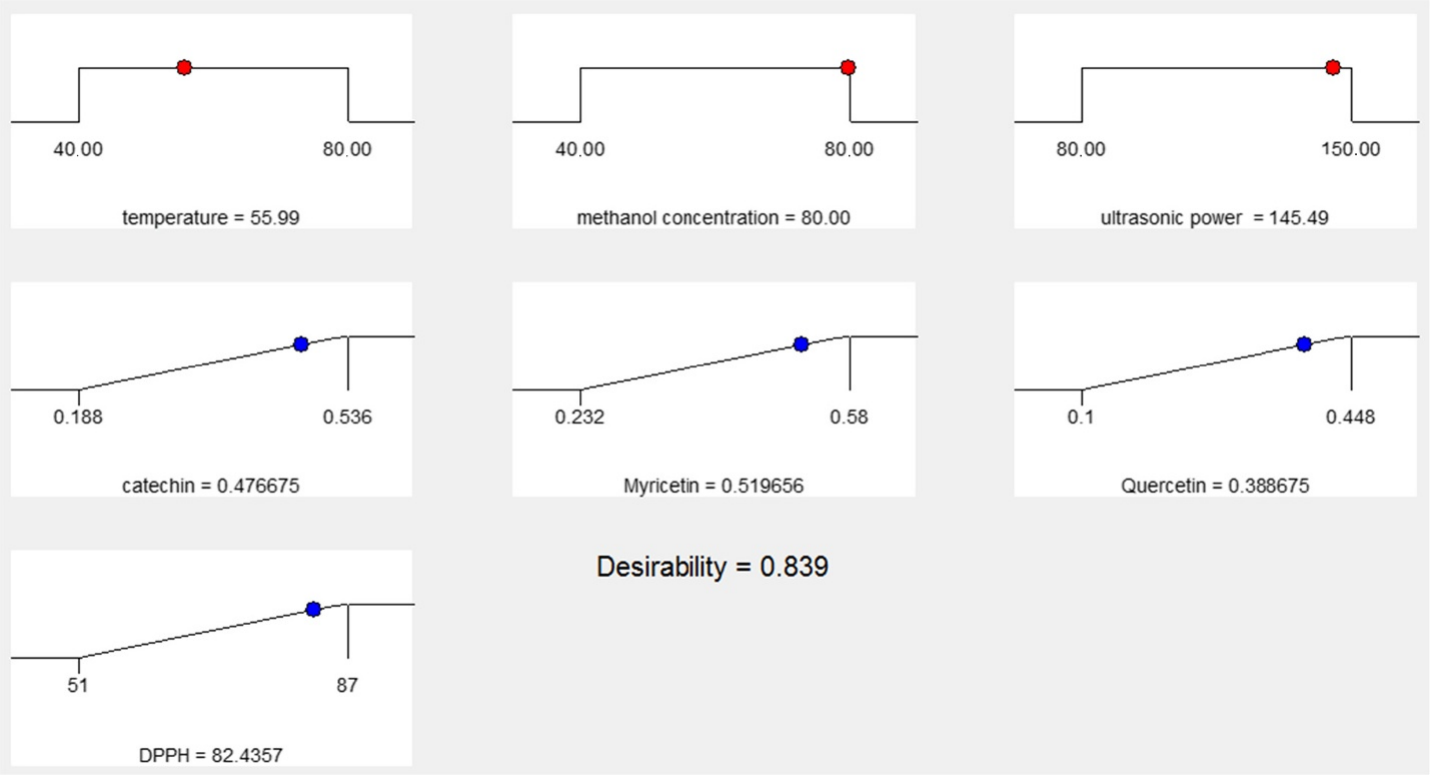
**The predicted optimization condition for reflux extraction of catechin, myricetin, quercetin and DPPH activity in curry leaf.**

Numerical optimizations and multiple graphical plots have been conducted in order to establish the optimum level of independent variables with desirable response goals. In the current study, for all responses only one optimal condition was obtained: 55.9°C temperature, 80% methanol, and 145.49 W ultrasonic power (Figure 3). After using the optimum conditions for extraction, the predicted values for catechin, narengine, quercetin, and the antioxidant activity were 0.476, 0.519, 0.388 mg/g DW, and 82.4%, respectively.

### Verification of the ultrasound-assisted extraction model

An experiment was conducted to verify the adequacy of the developed extraction model with the predicted optimum treatment conditions (55.9°C, 80% methanol, and 145.49 W). Under these conditions, the obtained concentration of catechin, narengine, quercetin, and the antioxidant activity were 0.482, 0.517, 0.394 mg/g DW, and 83%, respectively. The results of response surface analysis for catechin, myricetin, quercetin, and the antioxidant activity were verified by comparing the predicted values with the experimental values. The obtained results from verification experiment were in consent with the predicted values, because an insignificant (*P* > 0.05) difference was observed between the verification experimental and the predicted values.

### Evaluation of anticancer activity of optimized and unoptimized curry leaf extract

**Figure 4:**
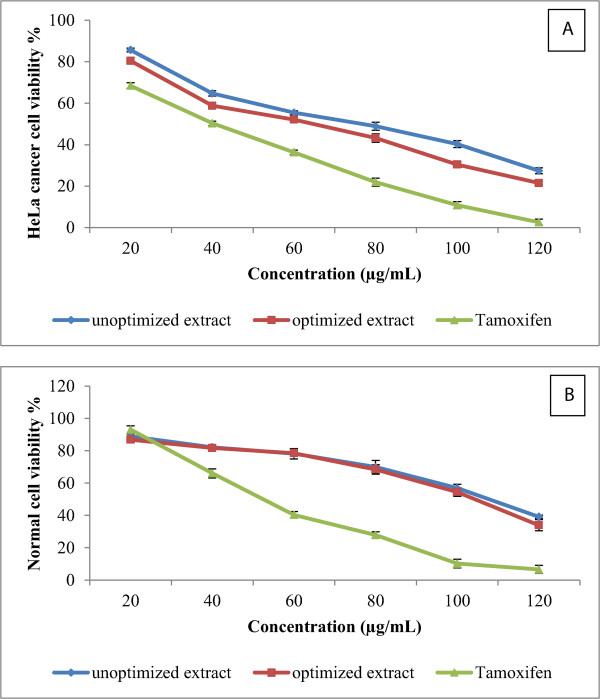
**Dose-dependent anticancer activity of curry leaf extracts against HeLa cell line (A) and normal cell viability (B).** Tamoxifen was used as a positive control. Bars represent standard error of means.

Optimized and unoptimized curry leaf extracts were used in order to evaluate their anticancer activity against HeLa cancer cell lines. Preliminary screening showed that the curry leaf extracts exhibited a significant anticancer activity against HeLa cancer cells at a concentration of 80 μg/mL, with an inhibition rate of 51.1% and 56.8% from the unoptimized and optimized extracts, respectively (Figure 4A). Tamoxifen (positive control) at a concentration of 80 μg/mL, showed 79.1% inhibition of HeLa cells. Furthermore, optimization of the reflux curry leaf extraction enhanced the anticancer activity by about 16.1%. The half maximal inhibitory concentration (IC_50_) values of the optimized and unoptimized extracts were found at concentrations of 67.2 and 81.8 μg/mL, respectively. As shown in Figure 3, the normal cells treated with the optimized and unoptimized extract of curry leaves showed 69.9% and 68.4% viability, respectively. According to the obtained results, optimized and unoptimized curry leaf extracts showed nontoxic effects at concentrations below 112 μg/mL, but toxic effects of the curry leaf extracts were observed at concentrations higher than that. Naik *et al.* [26] reported *M. koenigii* is more toxic to the mouse macrophages because of the high alkaloid content. The antioxidant and anticancer activities of the herbal extracts are directly related to phytochemicals or secondary metabolites of the extract [27, 28].

This study confirmed that anticancer activity of curry leaf extracts is associated with amount of potent flavonoid compounds because the highest anticancer activity was observed from the optimized extract, which contained higher amounts of flavonoids than the unoptimized extracts. Increased activation in the DPPH assay from the optimized extracts corroborates these earlier findings. The anticancer activity of curry leaf extracts against MDA-MB-231 and MCF-7 cell lines (breast cancer) has been reported previously [15, 29, 30]. The anticancer activity of curry leaves against HeLa cancer cells and the effective dose of this extract have been scarcely reported. Therefore, the findings of this current study could be useful for future studies.

The reduced cost of extraction is clearly advantageous for the proposed ultrasound-assisted extraction method in terms of time, energy, and the enhanced final yield [31]. Conventional procedures such as reflux and maceration are often time and energy consuming, and are generally not always interesting from the industrial point of view. Among the others, many advantages can be pointed out when taking into account the ultrasound-assisted extraction of flavonoids from curry leaves, including fast procedure, reduction of experimental cost (time needed, energy required, equipment size), and no need for any additional treatment or chemicals to complete the experiments.

## Conclusions

In this study, ultrasound-assisted extraction of catechin, narengine, and quercetin, and the antioxidant activity of curry leaf extracts were successfully optimized using RSM. The results indicate that the ultrasonic power, methanol dilution, and extraction temperature significantly affect the extraction yields of flavonoids with consequent enhancement of the antioxidant and anticancer activities of the extracts. The results can be easily explained by considering that both the temperature and methanol content have a positive effect on the solubility of flavonoids in the extraction solution. The ANOVA results revealed that the extraction temperature is the most significant factor influencing the response variables investigated. It was found that an ultrasonic power of 145.52 W at 55.9°C with 80% methanol was the appropriate condition for the extraction of catechin, narengine, and quercetin from curry leaves, with the consequent high anticancer and antioxidant activities. The optimized extract showed a more distinct scavenging activity against DPPH and significant anticancer activities against HeLa cancer cell lines at a concentration of 67.2 μg/mL, without toxicity to normal cells. Thus, it could be concluded that flavonoids of the curry leaves contributed to the anticancer activity of the extracts.
